# Use of a Terrestrial LIDAR Sensor for Drift Detection in Vineyard Spraying

**DOI:** 10.3390/s130100516

**Published:** 2013-01-02

**Authors:** Emilio Gil, Jordi Llorens, Jordi Llop, Xavier Fàbregas, Montserrat Gallart

**Affiliations:** Department of Agri Food Engineering and Biotechnology, Universitat Politècnica de Catalunya, Esteve Terradas 8, Campus del Baix Llobregat D4, 08860 Castelledfels, Barcelona, Spain; E-Mails: Jordi.Llorens.Calveras@upc.edu (J.L.); Jordi.Llop-Casamada@upc.edu (J.L.); Xavier.Fabregas@upc.edu (X.F.); Montserrat.Gallart@upc.edu (M.G.)

**Keywords:** drift, vineyard, LIDAR, drift test bench, air injection nozzles

## Abstract

The use of a scanning Light Detection and Ranging (LIDAR) system to characterize drift during pesticide application is described. The LIDAR system is compared with an *ad hoc* test bench used to quantify the amount of spray liquid moving beyond the canopy. Two sprayers were used during the field test; a conventional mist blower at two air flow rates (27,507 and 34,959 m^3^·h^−1^) equipped with two different nozzle types (conventional and air injection) and a multi row sprayer with individually oriented air outlets. A simple model based on a linear function was used to predict spray deposit using LIDAR measurements and to compare with the deposits measured over the test bench. Results showed differences in the effectiveness of the LIDAR sensor depending on the sprayed droplet size (nozzle type) and air intensity. For conventional mist blower and low air flow rate; the sensor detects a greater number of drift drops obtaining a better correlation (r = 0.91; p < 0.01) than for the case of coarse droplets or high air flow rate. In the case of the multi row sprayer; drift deposition in the test bench was very poor. In general; the use of the LIDAR sensor presents an interesting and easy technique to establish the potential drift of a specific spray situation as an adequate alternative for the evaluation of drift potential.

## 1. Introduction

Adequate deposition in the whole canopy according to the specifications of the treatment is one of the objectives of a pesticide application. Meanwhile spray drift continues to be a major problem in applying agricultural pesticides. Drift can cause crop protection chemicals to be deposited in undesirable areas with serious consequences [1]. Drift reduction and improvement of efficiency of pesticide application process is one of the goals of the 128/2009/CE European Directive for a Sustainable Use of Pesticides [2]. The imminent and mandatory establishment of National Action Plans by every European Union (EU) member will include the definition, establishment and quantification of buffer zones with quantitative information about drift potential of every sprayer and configuration. According to ISO 22866:2005 [3] drift is defined as “the quantity of plant protection product that is carried out of the sprayed (treated) area by the action of air currents during the application process”. In an orchard setting, this includes droplets which move horizontally through the orchard canopy and out the sides of the orchard, and droplets which are above the canopy (due to direct spraying into the air or diffusion up from the sprayed canopy) and move vertically into the atmosphere. Most drift involves droplets which move above the canopy for some or all of their pathways [4].

A realistic representation of spray drift could, for example, not only reveals a given percentile of the spray drift expected at a given distance from a field, but it could show the entire range of spray drift that might be observed, caused by different weather conditions or the equipment (nozzle type) [5]. Spray drift has been studied extensively [6,7], in a series of field trials and for many crops. The results from these studies are currently used in pesticide registration in the EU. Specifically, the 90th percentile of all measured “drift values” (the amount of drifted residues) is commonly applied in ecotoxicological risk assessments. The data include the variability of spray drift between different fields (field trials) and the variability within fields (different Petri dishes placed at the same distance from the field border). But, despite the wide variety of collected data, not all the scenarios can be identified. Spray drift is highly influenced by many factors that may be grouped [8] into one of the following categories: equipment and application techniques; spray characteristics; operator care and skill. Diverse methodologies [9–12] developed in the last years to evaluate and quantify the effect of different parameters involved in the process, in a big effort to define a spray classification, have always resulted in great variability due to the influence of environmental conditions.

In general, arrangement of field tests for drift measurement is very difficult and expensive. The ISO 22866:2005 norm defines the procedure to quantify drift during field tests, but this method is complex, time consuming and depends heavily on external conditions such as wind, being difficult to adopt and may have poor result repeatability. These facts, together with the need to maintain the spray track perpendicular to the wind direction make the arrangement of field tests a cumbersome and difficult process. Other researchers [13] have concluded that a sequence of experiments could last for several hours avoiding changing the line of measurements as long as the average wind deviation was in the range of ±30° from the original line.

But independent of the difficulties of field trial arrangements, the key problem in spray drift and dispersion assessment studies [14] has been the quantification of spray droplet concentration as it cannot be accurately extrapolated from point measurements to determine spatial dispersion [4]. It helps conclude that presently available direct and indirect methods of spray drift measurements were inadequate for measuring plumes of drifting aerosols. For these reasons, different authors have proposed different drift measurements, in an attempt to develop easy, repeatable and precise methods as an alternative to current standards. There are many methods available for sampling spray drift, and a great variety of estimates of spray drift have been published based on mathematical analysis [15], probabilistic estimations [5] or through the development of computational models based on indirect drift measurements [16–18]. In [19] the authors developed a drift prediction equation for reference spraying to predict the expected magnitude of sedimenting drift for various drift distances and atmospheric conditions. In [20] a new drift test bench for measurement of drift generated by a boom sprayer in a simpler and quicker way than the ISO 22866:2005 methodology was developed. The same device was successfully used by [21] to assess drift potential of a citrus herbicide applicator.

Sensor technology is an interesting alternative for drift evaluation purposes. Several studies [4,14,22] were carried out using Light and Detection Ranging (LIDAR) technology to measure drift. The authors of [23] used LIDAR to measure near-field pesticide spray movements in wing-tip vortices of a spray aircraft but not downwind drift. Stoughton *et al*. [24] adapted LIDAR technology to measure pesticide movement above an oak forest. The LIDAR system was found to be a highly useful spray plume movement measuring tool, as evidenced by the return images of spray material aloft for up to 2000 m downwind and well up into the mixing layer.

The specific scenario of spray processes in orchards is one of the most risky activities from the environmental point of view. In these cases, several researchers have selected LIDAR as an alternative device for drift measurement. In [4] a LIDAR system developed at the University of Connecticut was used to measure the concentration of small droplets in the air above an orange orchard canopy during and after the sprayer operation. The LIDAR sensor was able to measure and evaluate airborne drift differences between stable and unstable conditions. The authors of [25] developed a model to predict airborne drift according the target structure. The model utilizes LIDAR measurements of optical transmission to predict the characteristics of airborne drift of plant protection product's (PPP) leaving the target orchard at different growth stages and modified drift characteristic for different methods of dose adjustment. Good agreement was demonstrated between the measurements and predictions of drift from a semi-dwarf apple orchard at full-dose application rates. LIDAR systems have been used successfully to observe spray dispersion in stable [22] and unstable atmospheric conditions [26]. The technique has also been used for monitoring dispersion of smoke from forest fires [27]. In [28] a methodology to calibrate a scanning elastic backscatter LIDAR and extrapolate droplet point measurements in both space and time was developed.

The objectives of this research were to verify the use of a LIDAR sensor to measure the drift cloud during pesticide application in a vineyard and to study the effect of different working parameters (nozzle type, sprayer characteristics and air settings) on the total amount of liquid exceeding the target canopy.

## 2. Material and Methods

An experimental study was designed to characterize the amount of spray liquid that traversed the canopy during the spray application process over a vertical vine crop by using a Light Detection and Ranging (LIDAR) sensor system. Field trials were carried out in Castell del Remei, Lleida (N.E. Spain) in August 2011. The vine was a common Spanish trellis system (Royat) of var. Merlot vineyard with a 3 m distance between rows and 1.5 m between plants in the row (2,222 plants·ha^−1^). All field trials were carried out during the last week of July, coinciding with the BBCH 83 crop stage [29].

### 2.1. Sprayers Adjustment

**Figure 1.**
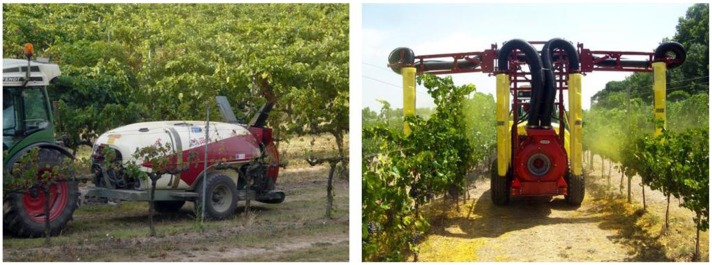
Conventional mistblower, Master 2000 (**left**) and multi-row sprayer, Iris-2 (**right**) used during the field trials.

The tractor (Fendt Farmer 207 DT) and the sprayer were driven parallel to the row at a constant forward speed of 4.4 km·h^−1^, in a straight line between the last two rows of vines. Two different sprayers, a conventional mist blower (Master 2000, Talleres Corbins, Lleida, Spain) equipped with a 940 mm diameter axial fan without deflectors and a multi row sprayer (Hardi Iris-2, Ilemo-Hardi, S.A.U., Lleida, Spain) with tangential turbine and individual oriented air outlets were tested (Figure 1). The conventional mistblower was adjusted using the two fan gear box (H and L), corresponding to 31.1 and 24.4 m·s^−1^ air speed, respectively. These two air speeds generated two different air flow rates of 34,959 m^3^·h^−1^ and 27,507 m^3^·h^−1^. These two air adjustments were combined with two different nozzle types: a conventional hollow cone ATR yellow, and air injection hollow cone, TVI-80015, both from Albuz (Saint-Gobain Ceramiques Advancees Desmarquest, Evreux, France). The droplet spectrum generated by these two nozzles has been classified as Very Fine (VF) and Coarse (C), respectively, according to [30,31]. The multi row unit was adjusted for an air flow rate of 6,423 m^3^·h^−1^ and was equipped with conventional hollow cone nozzles (Albuz ATR orange). Table 1 shows the details of the sprayer adjustments.

Air flow rate characteristics of the two sprayers were measured using a digital anemometer (Meteo Digit I, Lambrecht Klimatologische Messtechnik, Göttingen, Germany). Three replicates of the measurements were carried out at different points of the air outlets of the two sprayers (five points of measurement on each side of the air outlet of the conventional sprayer and 14 on each one of the four single drop legs for the multi-row sprayer) in order to obtain the average air speed (m·s^−1^) and its spatial distribution.

### 2.2. Spray Liquid and Tracer Concentration

Spray tanks were filled up to its half capacity with pure water and a certain quantity of a commercial tracer (Tartrazine, E-102, Sigma, St. Louis, MO, USA) was added in order to obtain a constant concentration of 8,000 mg·L^−1^. Each spray run consisted of a single pass of the tractor and sprayer between the last two rows of the parcel, simulating the generally established normal spray procedure. A total of three replicates were conducted for each test. Weather conditions during every test were recorded (Table 2) using an automatic weather station (WatchDog weather station Model 2550, Spectrum Technologies, Inc, Plainfield, IL, USA) placed 5 m away from the spray track.

### 2.3. Drift Detection Measurements

During the trials two different methods (LIDAR sensor and a test bench) were used to quantify the amount of spray liquid escaping the canopy and also its distribution over a perpendicular line away from the canopy. The two methods were used at a time during the tractor movement along the crop row.

**Figure 2.**
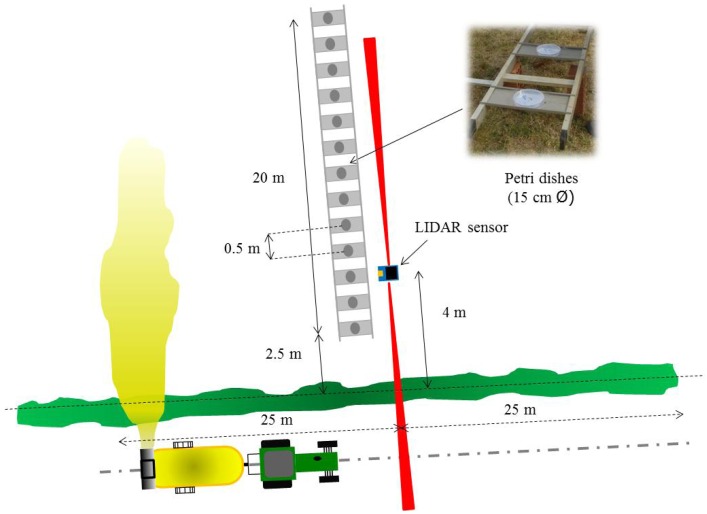
Scheme of placement of drift measurement devices (LIDAR sensor and test bench) related to last crop row and track followed by the tractor during field tests.

In the first method, drift measurements were made using a LIDAR sensor located at 4 m from the last sprayed canopy row, oriented to be able to measure the cloud drift on a perpendicular plane relative to the canopy row as shown in Figure 2. The LIDAR scanner used in this work was a low cost general-purpose (model LMS-200, Sick, Dusseldorf, Germany), with accuracy of ±15 mm and 5.2 mrad of divergence in a range up to 8 m, a selectable angular resolution of 1°, 0.5° or 0.25° and a scanning angle of 180°. This sensor has been previously used as electronic system for canopy characterization [32,33]. In this research, an angular resolution of 1° and a scanning angle of 180° were used. This setting allows obtaining a scan process of the whole area with gaps in between. Settings to get a full scan implicates to select 180° scanning angle with angular resolution of 0.5°, or 100° with 0.25 angular resolutions. In these cases, the LIDAR's manufacturer guarantee a complete scan but precision on time of measurements decreased considerably due to the limitations of the RS232 serial port. For that reason, those alternatives were rejected. The LMS-200 has a standard RS232 serial port for data transfer with a selectable rate of 9.6, 19.2 or 38.4 Kbit·s^−1^. For these assays the sensor was configured to record a scan cloud every 0.1 second.

**Figure 3.**
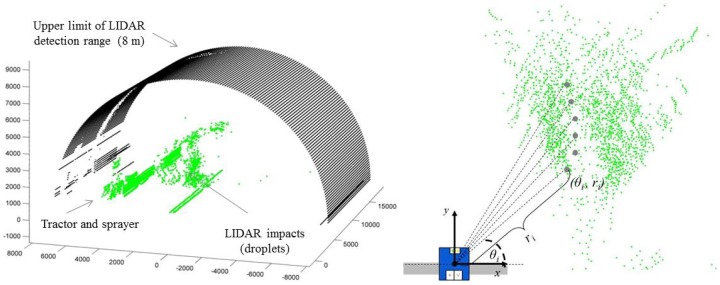
Example of LIDAR data plotted. Left part of the figure represents the three-dimensional view of the drift cloud escaping the canopy. Right part of the figure indicates the measurement process determining the values of angular position (*θ_i_*) and radial distance (*r_i_*) of e very single LIDAR impact.

**Figure 4.**
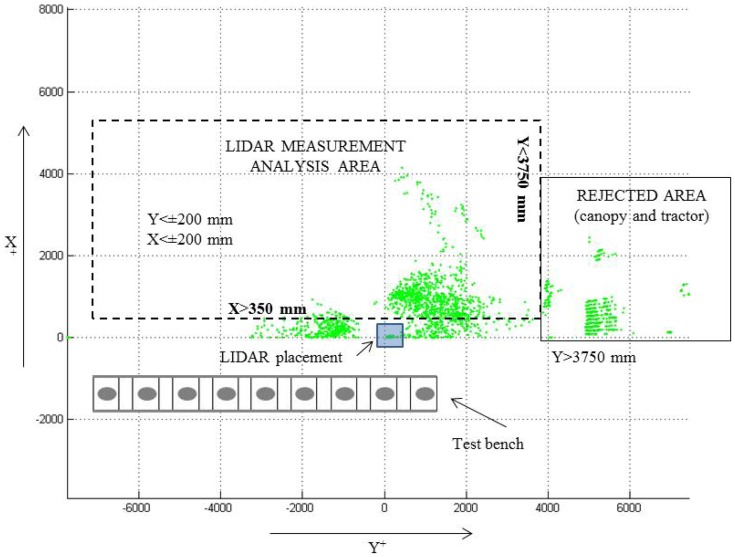
Example of LIDAR data plotted. Two-dimensional view of the drift cloud escaping the canopy where evaluation area is selected.

Spray drift cloud exceeding the canopy was scanned for an average of 40 seconds (total time of LIDAR scanning on a single test) during the spray track along the row, 20 s before the sprayer pass in front of the LIDAR and 20 s after, representing a total measurement distance of 50 m (Figure 2). When the drift cloud is intercepted by the laser beam, the sensor determines, from the reflected signal, the angular position *θ_i_* and the radial distance *r_i_* of every single impact (Figure 3), following the described process in [32]. The obtained data represents a vertical outline (or slice) of the drift for the current position of the LIDAR. When drift is produced, the LIDAR scanner supplied a cluster of impact points in 3D coordinates. Data acquisition process was arranged using the specific LIDARSCAN v.1® software (Universitat de Lleida, Lleida, Span), able to convert LIDAR impacts into data pairs according to *θ_i_* and *r_i_* parameters. MATLAB 7.11 software (The Mathworks Inc., Natick, MA, USA) was used for process support. This program allowed viewing a cloud of points in a plot of all the scans done during each trial and this program was used for all numerical analysis of data (Figures 3 and 4).

Drift was measured as the amount of liquid escaping the canopy. For this purpose, a kind of test bench was built. It consists of a 20 m long stain steel structure, placed perpendicularly to the canopy row, where artificial collectors (15 cm diameter Petri dishes) were placed at intervals of 0.5 m starting at 2.5 m away from the last canopy row and placed at 0.5 m over the ground (Figure 2). Petri dishes were picked up after the sprayer passes allowing collecting the spray liquid exceeding the canopy. Deposition on each sample was measured using a fluorimeter (Thermo Scientific Genesys 20, Waltham, MA, USA) after a dilution using a known amount of deionized water, following the methodology reported by [20,21]. Deposit on each artificial collector (*Di*), expressed in μL·cm^−2^, was calculated according to Equation (1):

(1) $${\text{D}}_{\text{i}}=\frac{\left[\left(\rho_{\text{smpl}}-\rho_{\text{blk}}\right)\times {\text{V}}_{\text{dil}}\right]}{\left(\rho_{\text{spray}}\times {\text{A}}_{\text{col}}\right)}$$

where: *ρ_smpl_* is the absorbance value (adim); *ρ_blk_* the absorbance value of the blanks (adim.); *V_dil_* the volume of diluent (deionized water) used to dissolve tracer deposit from collector in μl; *ρ_spray_* the absorbance value of the spray mix concentration applied during the tests and sampled at the nozzle (adim.); and *A_col_* is the projected area of the collector for catching the spray drift in cm^2^.

### 2.4. Relationship between LIDAR Data and Deposition on Test Bench

**Figure 5.**
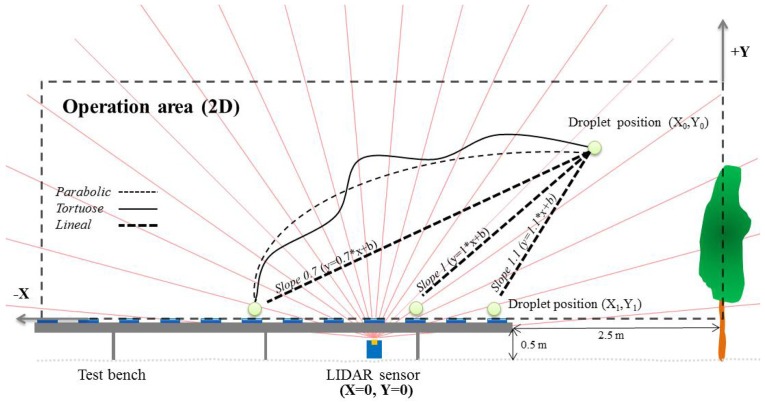
Representation of different droplet's trajectory applied to predict final deposit over the test bench. In this case the linear trajectory with slope value=1 was chosen to model the droplet's deposition.

This section describes the methodology developed for a comparative assessment of drift measures obtained with the two methods. A simple model based on a linear function was developed to estimate the droplet's trajectory measured with LIDAR sensor and its consequent deposit on a particular position over the test bench. Previous works have already modeled the droplets' trajectory using complex equations including a large number of parameters [16–19]. This aspect represented one of the most critical aspects of the whole process due to the difficulty in describing a drift model of droplet trajectory considering the great number of parameters involved [17]. However, for the purpose of this research, the most important parameter was the final position of the droplet in relation with the canopy position, independently of the trajectory (Figure 5).

The sedimentation model starts with the definition of a linear function. This function is then applied to the origin point (*X_0_*, *Y_0_*, Figure 5) in order to determine the final point (deposition point—*X_1_*, *Y_1_*). As the *X_1_* coordinate is a known value (the horizontal plane where collectors were placed, approx.: 350 mm), *Y_1_* can be calculated. After that procedure (*X_1_*, *Y_1_*) can be plotted on a graph, being necessary to repeat the whole process for all the points cloud detected on the cloud.

**Figure 6.**
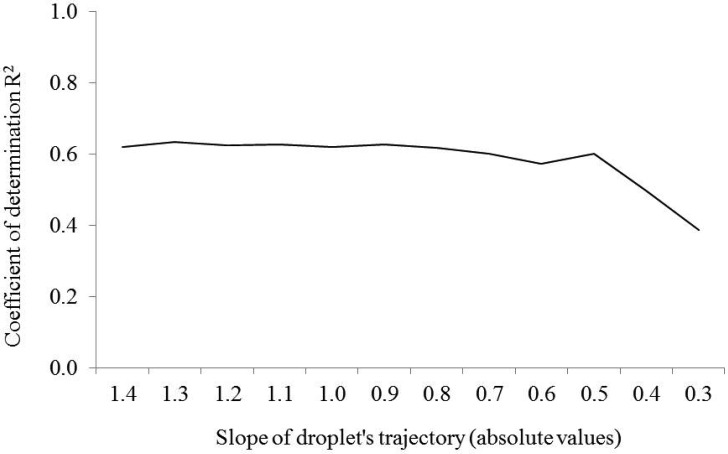
Relationship between average coefficient of determination (R^2^) from the regression analysis of all comparison among LIDAR drift data and test bench data, and the assumed negative slope of the hypothetical droplet's trajectory.

For that reason, a linear model was evaluated varying the slope value from −0.3 to −1.4 at 0.1 intervals and assessing in all cases the relationship with the distribution of deposits obtained with the test bench (Figure 6). Obtained results suggested good correlation without differences for slopes ranging from −0.9 to −1.4. According to that, the selected line slope value was −1.0, giving a compromise between the particular situations with high air flow velocity (higher adjustment with low line slope) and low air flow velocity (higher adjustment with high line slope).

## 3. Results and Discussion

### 3.1. Air Velocity and Air Profile of the Two Tested Sprayers

**Figure 7.**
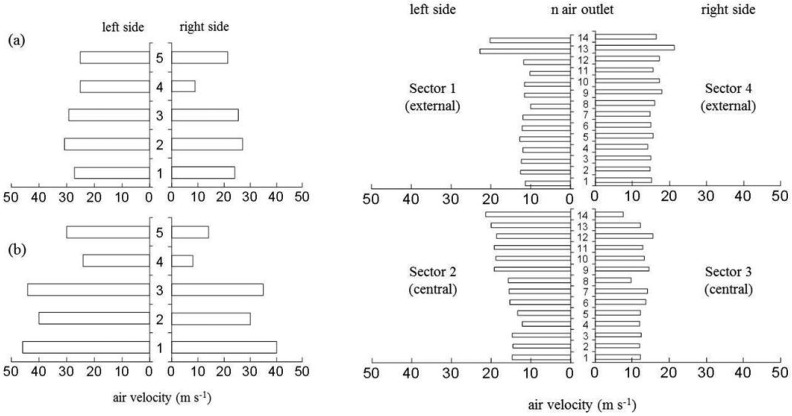
Air velocity profiles generated with the two sprayers. Left: conventional mist- blower at (**a**) low and (**b**) high air flow rate. Right: multi row sprayer at low air flow rate.

Averaged values of air velocity generated by the two sprayers were 31.1 m·s^−1^ and 24.4 m·s^−1^ for the two settings of the mistblower, and 14.6 m·s^−1^ for the multi-row sprayer. It is important to remark not only the great differences in terms of air velocity between the two tested sprayers, but also the uniformity of air distribution in all the air outlets. Figure 7 shows the air velocity distribution generated by the conventional sprayer at the two different tested conditions (high and low air flow rate) and the multi-row sprayer (low air flow rate). This figure indicates good uniformity of air distribution for multi-row sprayer, in comparison with the heterogeneity observed in the case of the mistblower, with great differences on air speed values depending on the measurement point of the air outlet, and also remarkable differences between left and right side, as a consequence of the fan rotation. Considerable improvements of air distribution were observed in the case of multi-row sprayer, where greater uniformity among the air velocity of the single air outlets was detected (Figure 7).

### 3.2. Determination of Drift Potential through LIDAR Impacts Evaluation

**Figure 8.**
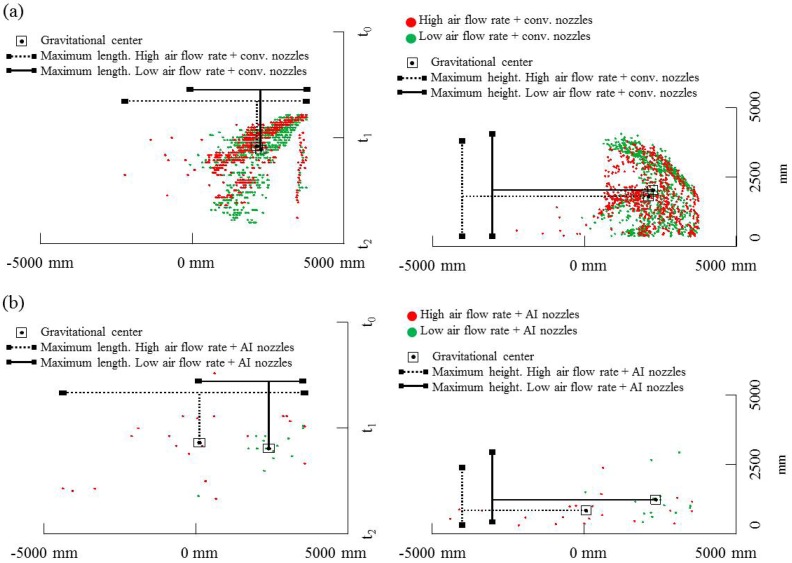
Example of zenithal (**left**) and elevation view (**right**) of drift cloud measured with LIDAR in different field tests: (**a**) drift cloud for conventional mist blower with conventional nozzles and using high (red) and low (green) air flow rate; (**b**) drift cloud for conventional mist blower with air injection nozzles and using high (red) and low (green) air flow rate. Maximum height, maximum length and gravitational center point are also represented for the LIDAR impacts' cloud. Note that 0 mm in X-axis represents the LIDAR placement.

**Figure 9.**
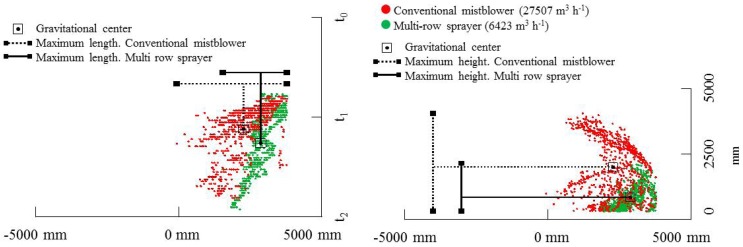
Plan (**left**) and elevation view (**right**) of drift cloud measured with LIDAR comparing conventional mistblower (red) and multi-row sprayer (green) both equipped with conventional nozzles and using low air flow rates. Maximum height, maximum length and gravitational center point are also represented for the LIDAR impacts' cloud Note that 0 mm in X-axis represents the LIDAR placement.

The placement of the LIDAR sensor in reference to the target area and the measurement procedure allows obtaining two different spatial estimations of drift escaping the canopy. Figures 8 and 9 show the spatial distribution of LIDAR impacts (potential drift) arranged according to the evaluated variable (type of sprayer, air flow rate and nozzle type). Figure 8 represents the comparison between drift cloud generated with the two tested air flow rates (red and green points respectively) and the effect of nozzle type (conventional or air injection nozzles), both represented in part (a) and (b) of the figure respectively. In all cases, left graphic plots the total LIDAR impacts from a zenithal view, during the whole spray time of 40 s (Y-axis) and covering the total sprayed row length (≈50 m). The right graphic in the figure represent the LIDAR impacts measured on a vertical plane from the point of sensor placement. This figure represents a cumulative measurement point of all single slices measured during the spray pass. In this case Y-axis indicates the drift cloud width measured from the sensor's placement during the total spray time (40 s).

Figure 8 also shows some indexes in order to assess the spray cloud generated by the LIDAR. Specifically it was calculate the maximum length, maximum height and gravitational center of the obtained LIDAR points. It is observed that total length of the drift cloud for the conventional mistblower when it was settled with high air flow rate and conventional nozzles was 2 m wider than that determined for low air flow rate with the same type of nozzles. No relevant differences (less than 0.3 m) were observed for the average drift cloud height and gravitational center placement. The effect of air injection nozzles can be observed in the lower part of Figure 8. In this case the air assistance generates a drift cloud twice as wide than the one detected with low air flow rate. In the case of high air flow rate LIDAR impacts were detected 8.0 m away from the canopy. Also in this case it is interesting to remark the differences in the gravitational center placement. High air assistance displaces the gravitational point far away from canopy.

Great differences on drift cloud depending on nozzle type can be observed. High impact density on the upper graphics on Figure 8 (conventional nozzles) could be assumed as an important portion of the spray exceeding the canopy, in comparison with the very low impact density obtained with air injection nozzles (lower part of the figure). But these differences can also be linked to the difficulty of the laser beam to impact on a less dense cloud, even if these droplets have bigger size (air injection nozzles) and, as a consequence, high amount of sprayed liquid close to the canopy. Figure 8 (part a) compares the drift clouds detected with LIDAR at different air flow rates. In this case the effect of high air assistance is clearly detected with LIDAR. Red points (corresponding to highest air flow rate) in both cases (horizontal and vertical plane plots) were detected far away from the canopy and in a most perpendicular position according to the target placement. This fact is related with the probability of finding spray deposit on soil at large distances from the canopy. The elevation view of the drift cloud (right part of the figure) shows the impact density of red points (high air assistance) at middle height, increasing the risk of droplets travelling far away from the target area. This tendency is also observed in the lower part of the figure, where values obtained with air injection nozzles has been represented. In this case, even for very low impact densities, the effect of air assistance on spray fraction far away of the intended target is clear.

It is important to remark that this research allows determining the spray cloud density, being difficult to equate a single LIDAR impact with a unique droplet [34]. In this case this study evaluates the spray cloud density and its relation with the total amount of liquid exceeding the target canopy.

Following the same structure previously explained, Figure 9 represents the comparative measurement of drift cloud for conventional sprayer at low air assistance (27,507 m^3^·h^−1^), and multi-row sprayer (6,423 m^3^·h^−1^). It is clearly observed that green points (which correspond to the multi-row sprayer) are much more concentrated close to the canopy (left part of the graphic corresponding to a zenithal view). This positive effect of lower risk of soil deposition far away of the canopy can be combined with less impact density from the multi-row sprayer (right part of the figure) which probably indicates a low spray amount escaping the canopy. As a consequence LIDAR measurements in this case represent an adequate tool/method to classify spray types according to its capacity to reduce drift.

The indexes obtained from LIDAR data also show in this case a clear effect depending on the sprayer type. The spray cloud is wider and higher in the case of the conventional mistblower than the one obtained with the multi-row sprayer. Also particular differences can be observed in the spatial placement of the gravitational center point of both drift clouds. Specifically, the placement of gravitational center point is 3.5 m above the ground level for the conventional mistblower, and 1.8 m above the ground for the drift cloud generated with the multi-row sprayer. This fact can be related with the potential risk of contamination of zones away from the sprayed area.

### 3.3. Deposition Curves with LIDAR and Test Bench

Following the procedure previously described, curves representing the distribution of spray deposits that have been obtained for the two proposed methods for drift measurement (test bench and LIDAR). Results obtained for every combination of working parameters (air adjustment, nozzle type, sprayer type) are compared in order to evaluate the most adequate method for drift measurements. Curves in pairs (LIDAR and test bench deposition) were compared by calculating the correlation coefficient (r). Table 3 shows the obtained values.

**Figure 10.**
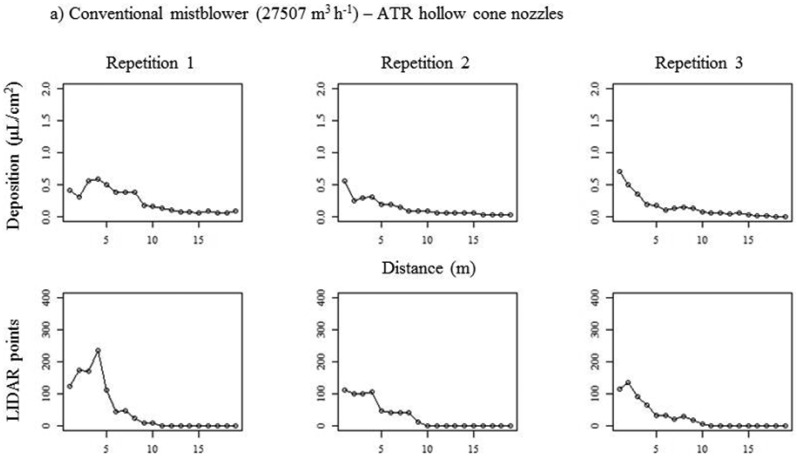

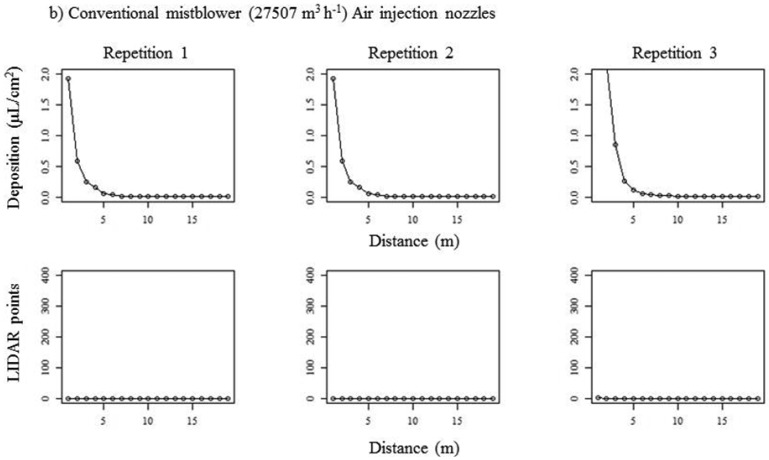
Curves representing deposition of spray deposit (test bench and LIDAR) obtained with conventional mistblower at low air flow rate. Part (**a**) corresponds to conventional hollow cones and part (**b**) corresponds to air injection nozzles.

Figure 10 represents the curves (three replicates) obtained with test bench and with LIDAR data after the application of the simulated droplet's deposition previously described.

The upper part of the Figure 10 (first to lines of graphics) corresponds to the conventional sprayer equipped with conventional hollow cone nozzles at low air flow rate. Good correlation between curves was obtained in the three cases, with values of the correlation coefficient (r) ranging from 0.85 to 0.94 (Table 3). In all cases, the biggest drift fraction was detected in the first 5 m of the measurement zone, close to the spray pass and canopy, and also it was observed a constant reduction of deposition after these 5.0 m. This reduction is much more intense in case of the LIDAR measurements.

The lower part of Figure 10 represents the spray deposit when air injection nozzles were mounted in the conventional sprayer, maintaining the low value of air flow rate. A detailed analysis of these graphics gives a clear indication of the high deposition measured in the collectors (test bench) placed close to the canopy (vine). In all cases (replicates) the deposition values went up to 2 μg·cm^−2^, higher than those obtained with conventional hollow cone nozzles but placed in all cases in the first 5 m from the canopy. The last row of graphics represents the translated LIDAR impacts to the test bench deposition using air injection nozzles. In all replicates a very low deposition was measured, being difficult to compare with values obtained with the test bench. This fact can be related with the previous explanation about the low LIDAR impacts obtained when sprayer was equipped with air injection nozzles, which can generate mistakes in the interpretation of the results, assuming low drift values when using high droplet sizes. It must be considered that big droplets contains higher spray liquid that the smaller ones. These difficulties in drift measurements using LIDAR have been previously reported (14, 28). However, LIDAR represents a good alternative for a quick and less time consuming drift evaluation processes.

**Figure 11.**
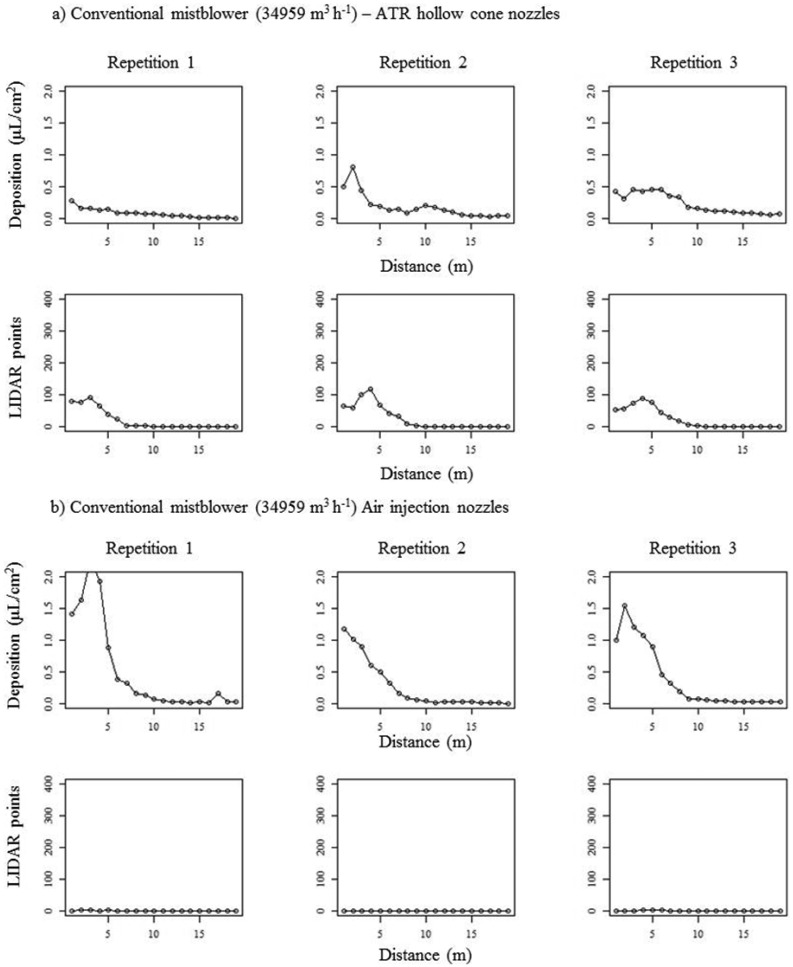
Curves of spray deposit (test bench and LIDAR) obtained with conventional mistblower at high air flow rate. Part (**a**) represents to conventional hollow cones and part (**b**) represents to air injection nozzles.

Figure 11 follows the same structure as described for Figure 10, representing, in this case, the results obtained with conventional mistblower at the highest air flow rate (34,595 m^3^·g·g·h^−1^). The effect of air flow rate on drift potential can be observed especially in the case of air injection nozzles (lower part of the graphic). Higher values of tracer deposit were found far away of the canopy placement as a consequence of the high air flow rate and air velocity, independently of the nozzle type installed on the sprayer. Also in this case, values of deposition obtained after LIDAR measurements were, particularly in the case of air injection nozzles, very low and not very well related with those obtained with the test bench. Again in this case, laser beam impacts were affected by droplet density. Correlation coefficients obtained after comparison of both drift measurement methods gave interesting values (from 0.60 to 0.87) with conventional hollow cone nozzles, also for high air flow rate. The worst correlation between the two proposed methods was observed again in the case of air injection nozzles, due to the difficulty of laser beam to detect less dense cloud of big droplets.

**Figure 12.**
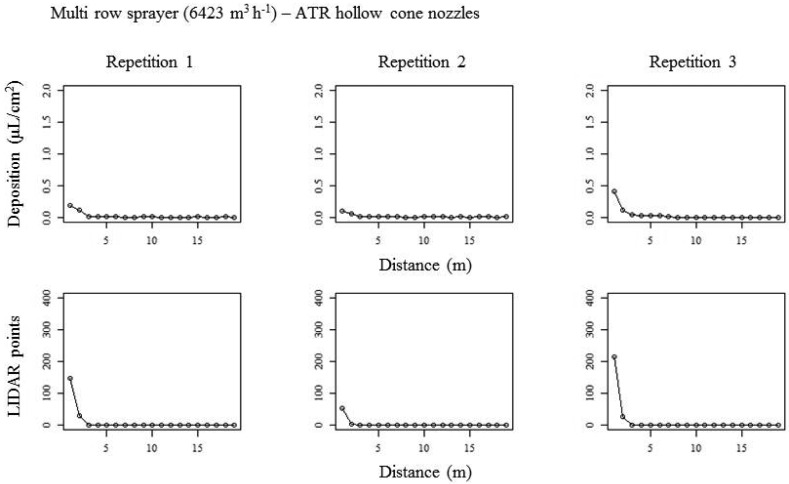
Curves of spray deposit (test bench and LIDAR) obtained with multi-row sprayer equipped with conventional hollow cone nozzles. Upper part of the figure represents the curves in test bench and lower part the curves obtained after LIDAR measurements.

Figure 12 shows a good relationship between the measured deposits obtained with the two proposed methods. In this case, as the spray liquid exceeding the canopy, using the multi-row sprayer, is expected to be lower than that generated by the conventional mistblower, both methods gave similar results. Also it is interesting to remark the high deposit measured in the area close to the canopy, as it was shown in Figures 8 and 9.

Following the main objective of this research, curves represented in Figures 10 to 12 indicate the predicted deposit of the spray cloud measured by LIDAR, being those compared with the real deposition values using artificial collectors.

Moreover, it is worth noting in this case the good correlation calculated between the two proposed methods, with values of correlation coefficient higher than 0.90 in all cases (Table 3). The interpretation of Figures 10, 11 and 12 must be done taking into account that LIDAR measurements allows to obtain values of impact's density, being those values linked to the amount of liquid exceeding the canopy target. Nevertheless, LIDAR measurements cannot be linked to droplet size [34].

## 4. Conclusions

In general the use of the LIDAR sensor represents an interesting and easy technique to establish the potential drift of a specific sprayer settings and environmental conditions. LIDAR system provides an idealized optical view of spray droplet escaping the canopy and its distribution away from the target. Furthermore, it allows to evaluating drift with less labor, cost and time than other current methods.

The use of test bench for drift measurement allows quantification of the amount of spray fraction escaping the canopy but the time required for the process is much higher than the one dedicated to LIDAR measurements.

In general, good correlation has been observed between the measured drift cloud with LIDAR and deposition distribution obtained on the artificial collectors placed in the test bench. However, it seems that drift measurements using LIDAR can be affected by droplet size.

The two proposed methods for drift measurement have shown potential in discriminating the effect of the different working parameters (nozzle type, air velocity and type of sprayer) on the drift. However, the results indicate a better ability of LIDAR sensor to evaluate spray drift in case of dense drift cloud. Additionally, further research should be arranged in order to assess the effect of sprayer's settings in the final droplet size in field conditions.

This technique will help the users to adjust an adequate deposition in the whole canopy according to the specifications of the treatment and could be used as a drift predictor tool depending on the target geometry, also in accordance with [25].

## Figures and Tables

**Table 1.** Sprayer settings during the field trials.

**Sprayer**	**Air flow**		**Nozzle type (n°)**	**Pressure (bar)**	**Droplet size** (1)	**Application rate**	
	
	**m·s^−1^**	**m^3^·h^−1^**				**L·min^−1 (2)^**	**L·ha^−1^**
Master 2000	24.4	27,507	ATR yellow (10)	8.0	VF	0.92	369
		27,507	TVI 80015 (10)	8.0	C	0.98	393
Master 2000	31.1	34,959	ATR yellow (10)	8.0	VF	0.92	369
		34,959	TVI 80015 (10)	8.0	C	0.98	393
Iris-2	14.6	6,423	ATR orange (16)	8.0	VF	1.24	398

(1) According to BCPC classification [30] (VF: Very Fine; C: Coarse);

(2) Flow rate per single nozzle.

**Table 2.** Weather conditions recorded during the field tests. RH: relative humidity; TMP: temperature; WND: wind direction; WNG: wind gust; WNS: wind speed; DEW: dew point.

**Test**	**Nozzle Type**	**Air Flow**	**Rep**	**RH (%)**	TM (*C)	**WND (°)**	**WNG km·h^−1^**	**WNS km·h^−1^**	**DEW °C**
Master 2000	ATR	Low	1	78.6	20.2	149	0	0	16.4
			2	81.6	19.7	190	0	0	16.5
			3	81.9	19.8	158	0	0	16.6
	TVI	Low	1	78.6	20.8	158	0	0	17.0
			2	76.5	21.1	84	0	0	16.9
			3	75.7	21.6	46	1	0	17.2
	ATR	High	1	71.7	22.5	83	0	0	17.2
			2	69.1	23.1	83	0	0	17.2
			3	68.0	23.8	66	0	0	17.7
	TVI	High	1	64.5	24.5	74	0	0	17.5
			2	62.1	24.6	83	0	0	17.0
			3	59.0	26.4	70	0	0	17.9
Iris 2	ATR	Low	1	41.1	30.6	46	1	0	15.9
			2	36.0	31.2	103	1	1	14.3
			3	33.9	31.5	115	3	3	13.6

**Table 3.** Values of correlation coefficient (r) between the number of points detected by LIDAR and the deposition of Tartrazine in the artificial collectors placed in the test bench.

**Sprayer Type**	**Air Flow Rate (m^3^·h^−1^)**	**Nozzle Type**	**Correlation Coefficient (r)**		

			**Rep 1**	**Rep 2**	**Rep 3**
Conventional	34,959	Conventional	0.87	0.60	0.91
		Air injection	0.88	0.32	0.40
Conventional	27,507	Conventional	0.85	0.91	0.94
		Air injection	0.07	0.73	0.88
Multi row	6,423	Conventional	0.93	0.91	0.98
